# Supplementary material for: Additively Manufactured Flexible EGaIn Sensor for Dynamic Detection and Sensing on Ultra-Curved Surfaces
Source: Sensors (Basel). 2024 Dec 25;25(1):37. doi: 10.3390/s25010037 (PMC11722807; doi:10.3390/s25010037)
Supplement: Supplementary file 1 [file sensors-25-00037-s001.zip › sensors-3336665-supplementary.pdf]

Supporting Information

Additively manufactured flexible EGaIn sensor for dynamic detection and sensing on ultracurved surfaces

Jiangnan Yan† [1,2], Jianing Ding† [1,2], Yang Cao† [1,2], Hongyu Yi[1,2], Yifan Gao [1,2], Kongyu Ge [1,2], Hongjun Ji [1,2], Mingyu Li [1,2], Huanhuan Feng [1,2,*]

1.Sauvage Laboratory for Smart Materials, Shenzhen Key Laboratory of Flexible Printed Electronics Technology, Harbin Institute of Technology, Shenzhen 518055, China

2.State Key Laboratory of Advanced Welding and Joining (Shenzhen), Harbin Institute of Technology, Shenzhen 518055, China

*Correspondence: fenghuanhuan@hit.edu.cn; 18682368507

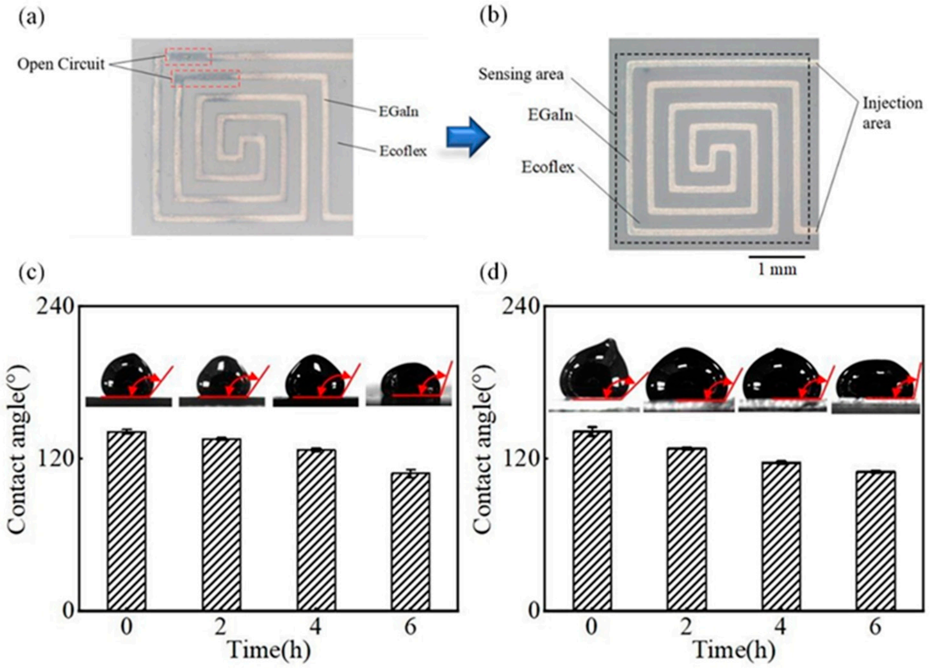

Figure S1: Image of EGaIn flexible sensor. (a) Original EGaIn flexible sensor; (b) Modified EGaIn flexible sensor.

Contact angle evolution of EGaIn on different material surfaces over time of oxidation: (c) Ecoflex; (d) PDMS.

We modified EGaIn by a relatively simple oxidation control method, appropriately reducing its surface tension, thereby enabling the millimeter-scale electronic skin to exhibit more stable and reliable performance. Specifically, the original EGaIn was mixed with a 0.2 mol·L^{-1} NaOH solution (24 g of EGaIn and 20 mL of NaOH solution). After undergoing ultrasonic cleaning for 10 minutes to remove oxides, the mixture was stirred with a magnetic stirrer at 200 r/min for 4 hours to induce oxidation. The modified EGaIn demonstrated improved wettability and reduced surface tension, ensuring successful filling of the microchannels (Figure S1.b)

To further demonstrate the improvement in wettability and adhesion properties due to oxidation, we visually measured the contact angle of EGaIn on Ecoflex and PDMS as shown in Figure S1.c and d. Before oxidation, the contact angles of EGaIn on Ecoflex and PDMS were 140.9° and 141.2°, respectively. With continuous stirring and oxidation, the contact angles exhibited a significant decrease. After 6 hours of stirring, the contact angles decreased to 108.1° on Ecoflex and 109.3° on PDMS. Oxidizing EGaIn markedly enhanced its wettability and adhesion on both Ecoflex and PDMS surfaces. However, prolonged oxidation increased the overall viscosity of EGaIn, reducing its flowability to a paste-like consistency. Therefore, for fabricating millimeter-scale electronic skin, we used EGaIn oxidized for 4 hours to fill microchannels.

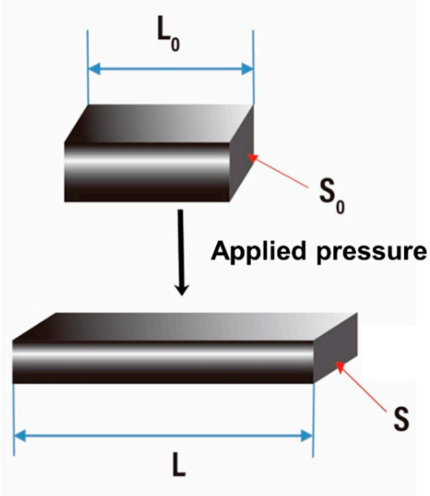

Figure S2: Theoretical model of metal liquid deformation with applied pressure

Our sensor identifies applied pressure through the deformation of microchannels. The change in sensor resistance is primarily due to the variation in the resistance of the liquid metal circuit. When pressure is applied to our sensing unit, the cross-sectional area of the microchannel decreases, leading to a corresponding change in resistance (Figure S2). This resistance variation follows the theoretical calculation as described below:

$$\Delta R = \frac{\rho L}{wh}\left\{\frac{1}{1-2(1-v^2 w\chi p/Eh)}\right\}$$

From:

Resistance law:

$$R=\rho\ L/S$$

Volume formula:

$$S=V/L$$

And,

$$\frac{R}{R_0} = \left(\frac{L}{L_0}\right)^2$$

To:

$$\boldsymbol{R = R_0(1+\varepsilon)^2}$$

where v is the Poisson's ratio of Ecoflex; E is the elastic modulus of Ecoflex; p is the pressure applied to the sensor; L, w, and h are the length, width, and height of the sensor circuit channel, respectively; and χ is the correction constant.

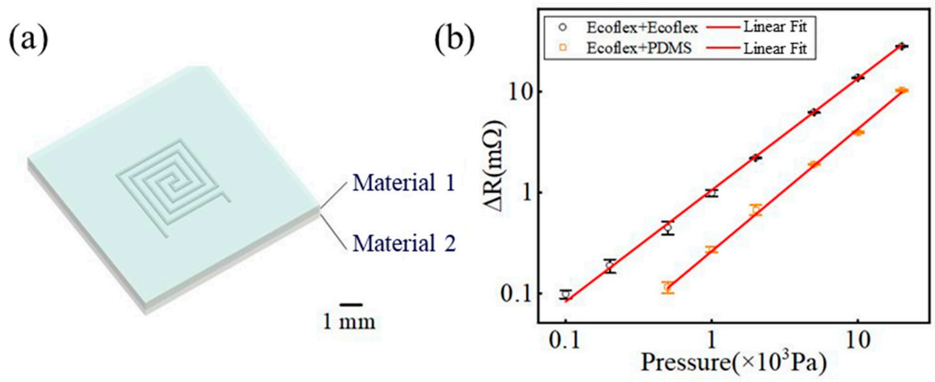

Figure S3: Sensor Unit Model and Comparison of Linear Sensing Ranges Among Sensor Units with Different Substrates

(a) Model Diagram of the Sensing Unit (b) Comparison of Linear Sensing Range between Sensing Units with Different Substrates.

Using the mold-releasing method, we obtained more precise, millimeter-scale electronic skin with smaller dimensions. However, this change in the preparation method also introduced a new material, PDMS, into the flexible substrate of the electronic skin. PDMS offers a wide process window for achieving double-layer bonding, easy control of its semi-cured state, and a high bonding success rate. Yet, the change in the preparation material is bound to have a certain impact on the sensing performance of the sensing unit. Therefore, we conducted a comparative study using controlled experiments. The actual model diagram of the prepared sensing unit is shown in Figure S3.a, where the combinations labeled as "Material 1 + Material 2" during actual preparation correspond to "Ecoflex + Ecoflex" and "Ecoflex + PDMS," respectively, with the conductive material filled being EGaIn oxidized for 4 hours. We tested the linear sensing range and pressure resolution of the two prepared sensing units for comparative research.

Firstly, we determined the linear sensing range of the sensing units with two different substrate materials through independent weight loading experiments, yielding the results shown in Figure S3.b. By comparison, it can be seen that when the applied pressure is less than 500 Pa, the sensing unit with "Ecoflex + PDMS" as the flexible substrate cannot produce a resistance response; when the applied pressure is in the range of 500-20000 Pa, although the "Ecoflex + PDMS" sensing unit produces a resistance response, the incremental resistance response is also much lower than that of the sensing unit with "Ecoflex + Ecoflex" as the flexible substrate under the same pressure conditions. By comparing the response results, we can find that the introduction of PDMS significantly narrows the linear sensing range of the sensing unit and degrades its sensing performance. Then, we determined the pressure resolution of the sensing units with two different substrate materials through step-by-step loading experiments, with the results shown in Figure S3.c. The step-by-step loading curves indicate that under the same pressure conditions, the incremental resistance produced by the "Ecoflex + PDMS" sensing unit is significantly smaller than that of the "Ecoflex + Ecoflex" sensing unit, and the minimum resolvable pressure loading also changes from 20 Pa to 100 Pa. The introduction of PDMS also degrades the pressure resolution of the sensing unit. These two aspects of degradation can essentially be classified as one type, namely, the weakening of the resistance response of the sensing unit when pressure is applied, to the point that the originally resolvable pressure loading becomes indistinguishable due to the weakened response. This phenomenon occurs because the Young's modulus of PDMS is 2.4 MPa, while the Young's modulus of Ecoflex is 0.13 MPa. PDMS has weaker deformation ability than Ecoflex, and the overall deformation ability of the flexible substrate is reduced after the introduction of PDMS. When subjected to the same pressure load, the deformation of the flexible substrate and EGaIn decreases, leading to a decrease in the resistance response. Therefore, under conditions

permitted by the preparation process, using materials with superior deformation ability to prepare the flexible substrate can enable the sensing unit to achieve more excellent sensing performance.

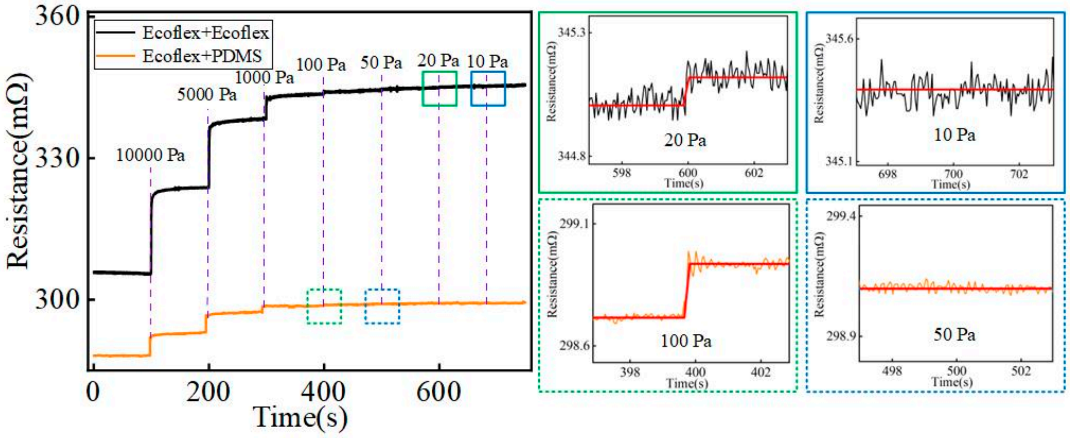

Figure S4: Comparison of Pressure Resolution between Sensing Units with Different Substrates.

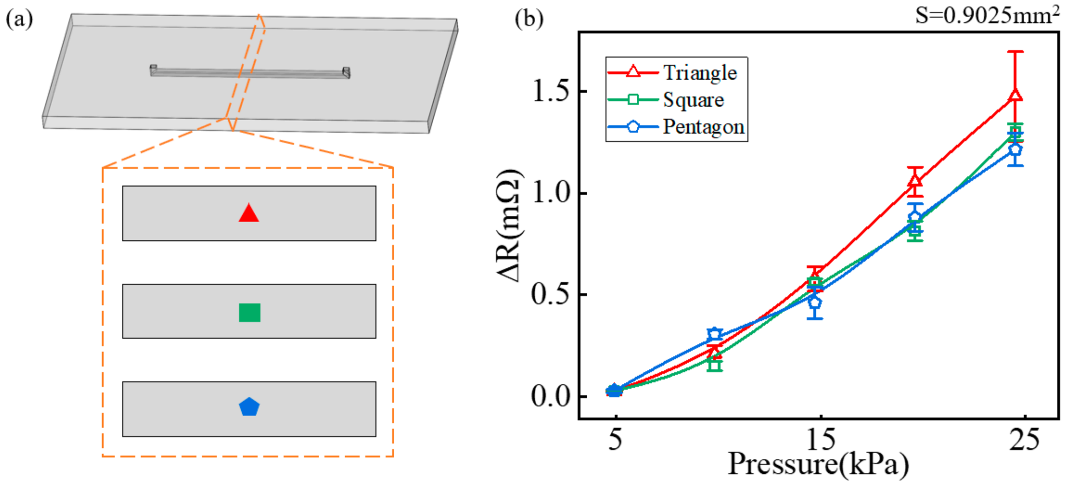

Figure S5: Models and Response Results of Samples with Different Cross-Sectional Shapes

For sensor devices with a cross-sectional area of 0.9025 mm^2 and conductive pathways in the shapes of triangles, squares, and pentagons, there are slight differences among them overall, but the actual sensing performance does not vary significantly. Considering the need to further reduce the channel size in the future, the more regular shape of the square is more suitable.

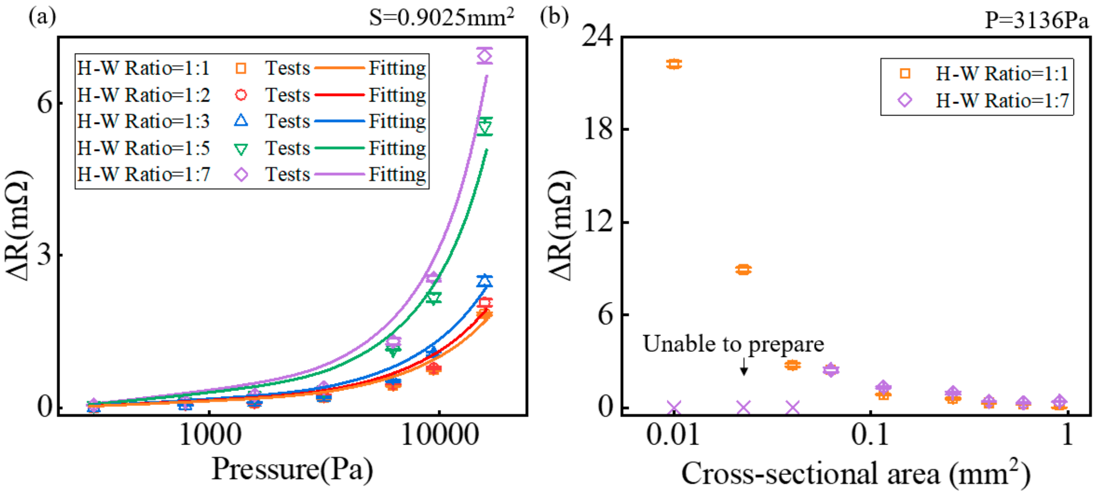

Figure S6: Impact of Different Aspect Ratios and Cross-Sectional Sizes on Resistance Response

Under the same pressure loading conditions, devices with a smaller aspect ratio exhibit higher sensitivity. The device with an aspect ratio of 1:7 has the highest sensitivity. Although the 1:7 device shows the highest sensitivity, it is challenging to further fabricate devices with such an aspect ratio after reducing the cross-sectional area due to machine limitations. Therefore, samples with an aspect ratio of 1:1, made with a reduced cross-sectional area, demonstrate superior sensitivity. Additionally, this "flat" shape tends to cause significant shape distortion during molding, and it is also not suitable for extending the length of the conductive pathway within a limited area.

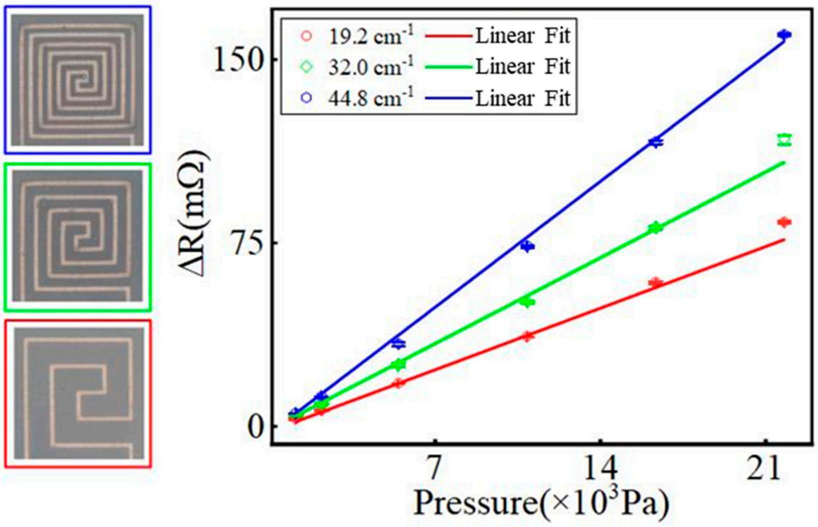

Figure S7: Impact of Different Wiring Densities on Resistance Response

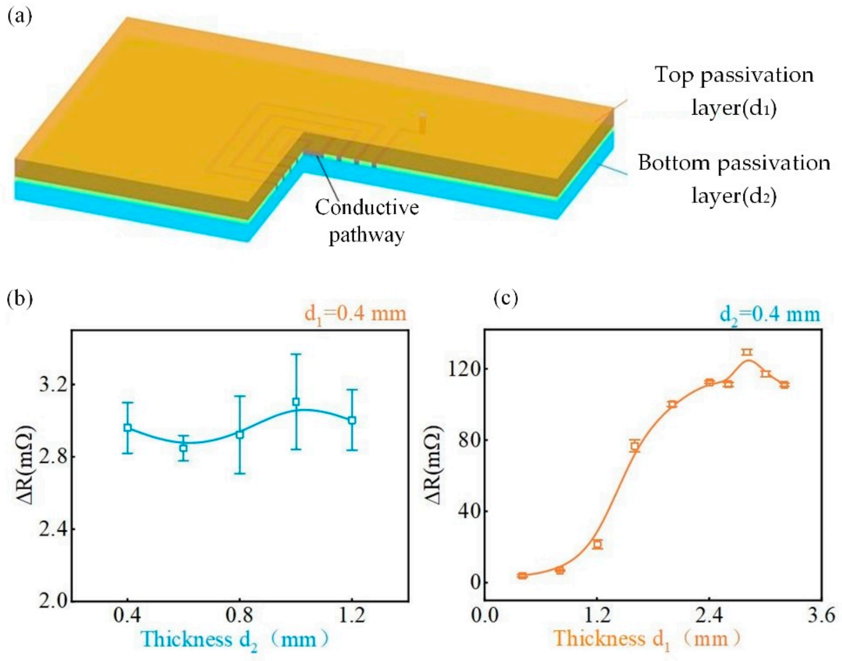

Figure S8: Exploration of Sensor Unit Model and the Impact of Passivation Layer Thickness on Sensing Performance. (a):Model Diagram of the Sensing Unit;(b)Impact of Bottom Passivation Layer Thickness on Sensing Performance; (c)Impact of Top Passivation Layer Thickness on Sensing Performance

Initially, we fixed the thickness of the top passivation layer (d_1) at 0.4 mm and varied the thickness of the bottom passivation layer (d_2), obtaining the resistance response of the sensing unit to a 20,000 Pa pressure load as shown in Figure S8(b). The results indicate that for the various thicknesses of the bottom passivation layer set, due to the relatively poor deformation capability of PDMS, the resistance increment caused by pressure loading fluctuated within a small range around 3.00 mΩ, and changes in the thickness of the bottom passivation layer had essentially no impact on the sensing sensitivity of the unit. Therefore, we set the bottom passivation layer thickness (d_2) to 0.4 mm and changed the thickness of the top passivation layer to further study its impact on sensing sensitivity, with the response results shown in Figure S8 (c). From the response results, we found that as the thickness of the top passivation layer increased, the resistance increment of the sensing unit first increased and then decreased, showing the largest resistance increment and highest sensitivity when the top passivation layer thickness reached 2.8 mm. Thus, the bottom passivation layer made of PDMS has essentially no impact on the sensing sensitivity of the unit, and appropriately increasing the thickness of the Ecoflex top passivation layer helps achieve higher sensing sensitivity and superior sensing performance.
